# Identification of Differential Expression Genes in Leaves of Rice (*Oryza sativa* L.) in Response to Heat Stress by cDNA-AFLP Analysis

**DOI:** 10.1155/2013/576189

**Published:** 2013-02-17

**Authors:** Yunying Cao, Qian Zhang, Yanhong Chen, Hua Zhao, Youzhong Lang, Chunmei Yu, Jianchang Yang

**Affiliations:** ^1^College of Life Science, Nantong University, Nantong, Jiangsu 226019, China; ^2^Key Laboratory of Crop Genetics and Physiology of Jiangsu Province, Yangzhou University, Yangzhou, Jiangsu 225009, China

## Abstract

High temperature impedes the growth and productivity of various crop species. To date, rice (*Oryza sativa* L.) has not been exploited to understand the molecular basis of its abnormally high level of temperature tolerance. To identify transcripts induced by heat stress, twenty-day-old rice seedlings of different rice cultivars suffering from heat stress were treated at different times, and differential gene expression analyses in leaves were performed by cDNA-AFLP and further verified by real-time RT-PCR. In aggregate, more than three thousand different fragments were indentified, and 49 fragments were selected for the sequence and differential expressed genes were classified functionally into different groups. 6 of 49 fragments were measured by real-time RT-PCR. In addition, the variations of three different polyamine contents in response to heat stress through high-performance liquid chromatography (HPLC) analysis were also performed. The results and their direct and indirect relationships to heat stress tolerance mechanism were discussed.

## 1. Introduction

With the development of industrialization, the impact of ongoing climate change on the natural environment deterioration was more and more obviously shown. Due to an increase in mean global temperature, heat stress has become a major disastrous factor that severely affects the crop cultivation and productivity. Heat stress-induced decrease of the duration of developmental phases leading to fewer organs, smaller organs, reduced light perception over the shortened life cycle, and perturbation of the processes related to carbohydrate metabolism (transpiration, photosynthesis, and respiration) is most significant for losses in cereal yields [1]. 

 Being a staple food and a principal calorie source for people living in Asia [2], rice (*Oryza sativa *L.) production and its factors has attracted more and more attention. However, rice is also a prime example of cereal crops whose growth and reproduction could be impaired by heat stress. It has been indicated that rice production will be severely affected by the aberrant change of temperature [3]. Comprehending the mechanisms of which rice response to heat stress would facilitate the development of heat-tolerant cultivars with enhanced productivity in a warmer future climate. The researches that underlie molecular responses both in genomics and proteomics level of plants to heat stress have, therefore, attracted a great deal of attention [4–6].

 Previous proteomic analysis with the employment of various advanced techniques such as two-dimensional electrophoresis provides a cogent approach to investigate the molecular mechanisms of rice response to abiotic stresses [7, 8]. Relative proteomic studies about rice in response to abiotic stresses have been deep into subcellular proteome and posttranslational modifications [7]. However, it is well known that the expression of proteins is regulated by the gene transcription and translation processes, although proteomic study about rice in response to heat stress is significant, the assessment of gene level regulation of such type of temperature variability on rice is also unneglectful. In addition, rice may confront with different high-temperature stresses during its lifespan, and different growth phases may have different responses to the stresses, which will be inevitably regulated by the variation of the expression of genes. Previous proteomic studies revealed that seedlings-phased rice could also be affected by the heat stress environment [9]. Although genomewide expression analysis showed that transcription level of heat shock proteins (Hsps) and heat shock factors (Hsfs) are induced under heat stress at young seedling stages [10, 11], further investigations to uncover the regulative mechanisms corresponding to heat tolerance at genomics level are still necessary.

 Among techniques employed in gene identification, many techniques such as differential display reverse transcription-polymerase chain reaction (DDRT-PCR), representational difference analysis (RDA), serial analysis of gene expression (SAGE), suppression subtractive hybridization (SSH), and cDNA microarray provide effective approaches for transcription analysis [12]. As a high-efficient, less labor-intensive mRNA fingerprinting method for isolation of differentially expressed genes [13], cDNA-amplified fragment length polymorphism (cDNA-AFLP), which has been employed in the study, is a robust genomewide expression tool [12] for gene discovery without a prerequisite of the prior knowledge of the sequences [14]. In addition, due to its high detective sensitivity of cDNA-AFLP analysis, some rare transcripts could also be detected by this method [15]. This technique has been further ameliorated to avoid the possibility of several transcript-derived fragments (TDFs) from a single gene/cDNA [16].

 Currently, several rice varieties defined by temperature thresholds [17] have been identified with genotype variation in spikelet sterility at high temperature in *indica* and *japonica* [18, 19]. In our study, forty-nine critical genes differentially expressed in rice seedling in response to heat stress using cDNA-AFLP technique have been identified. To validate their expression patterns, six gene fragments involving different physiological activities were analyzed through real-time PCR. In addition, due to direct interactions of polyamines with other metabolic pathways during the stress response [20] and their regulatory functions in plant abiotic stress tolerance [21], the variations of three different polyamine contents in response to heat stress through high-performance liquid chromatography (HPLC) analysis were also performed. These outputs may lay the fundamental basis of the rice variety breeding in the future. 

## 2. Materials and Methods

### 2.1. Plant Material

Seeds of two* indica* rice cultivars (Shuanggui 1, heat sensitive; Huanghuazhan, heat tolerant) were provided by the Rice Research Institute of Guangdong Academy of Agricultural Sciences (Guangzhou, China). The seedlings were grown in Kimura B complete nutrient solution, under greenhouse conditions (28°C day/22°C night, relative humidity 60–80%, the light intensity 600–1000 *μ*mol m^−2^ s^−1^, and photoperiod of 14 h day/10 h night), with 50 seedlings per pot. Then, twenty-day old seedlings were heat-stressed (at 39°C day/30°C night) for two different treatment times: 24 h or 48 h, while control seedlings were grown under the conditions mentioned above. Each treatment had 3 pots as replications. Samples from rice seedling leaves were harvested and were immediately frozen in liquid nitrogen and stored at −80°C until use. Heights of rice seedlings of Shuanggui 1 at 28°C control condition or 39°C stress condition were determined with a ruler from the land surface to the top of the seedling leaves with means of three replications (50 plants per replication).

### 2.2. RNA Extraction

Total RNA was extracted using RNeasy Plant Mini Kit (QIAGEN) according to the manufacturer's instructions. The integrity of the RNA was checked by agarose gel electrophoresis and the concentration was determined at 260 nm by spectrophotometer. 

### 2.3. cDNA-AFLP Analysis

An cDNA-AFLP method was adapted from Bachem et al. [13, 22] with a few modifications. Synthesis of double-stranded cDNA was performed with M-MLV RTase cDNA Synthesis kit (TaKaRa, China) and refined using phenolchloroform extraction. 5 *μ*L was checked using agarose gel electrophoresis in order to observe an expected smear between 100 bp and 1000 bp. The rest of cDNA was digested using the restriction enzymes *Eco*R I (Fermentas; 2 h at 37°C) and *Mse* I (*Tru*1I, Fermentas; 2 h at 65°C). The digested products were ligated to adaptors (*Eco*R I, 5 *μ*mol L^−1^, forward primer: 5′-CTCGTAGACTGCGATCC-3′, reverse primer: 5′-AATTGGTACGCAGTCTAC-3′;* Mse* I, 50 *μ*mol  L^−1^, forward primer 5′-GACGATGAGTCCTGAG-3′, reverse primer 5′-TACTCAGGACTCAT-3′) with T4-DNA ligase (Fermentas) for 13 h at 16°C. The products of ligation were amplified with the corresponding preamplification primers (*Eco*R I: 5′-GACTGCGTACCAATTC-3′; *Mse *I: 5′-GATGAGTCCTGAGTAA-3′). Preamplification was initiated at 94°C for 3 min and followed by 30 cycles at 94°C for 30 s, 56°C for 30 s, 72°C for 1 min, and terminated at 72°C for 5 min. The products of the preamplification were checked by agarose gel electrophoresis (expected smear between 100 bp and 1000 bp). From a 20-fold dilution of the pre-amplified samples, a 5 *μ*L sample was used for the final selective amplifications using the primers 5′-GACTGCGTACCAATTCNN-3′ (*Eco*R I, NN represents AC, AG, GA, and GT) and 5′-GATGAGTCCTGAGTAAMM-3′ (*Mse* I, MM represents TC, TG, CA, and CT). After initial denaturation (94°C for 3 min), 12 cycles were performed with touchdown annealing (94°C for 30 s, 65°C to 56°C in 0.7°C steps for 30 s, 72°C for 1 min) followed by 24 cycles (94°C for 30 s, 56°C for 30 s, 72°C for 1 min) and final elongation (72°C for 5 min). Altogether 64 primer combinations of two base extensions (denoted as NN and MM) were used.

### 2.4. Polyacrylamide Gel Electrophoresis

 The selective amplification products were separated on a sequencing polyacrylamide gel (6% polyacrylamide, 8 mol L^−1^ urea, 1×TBE) at a constant current mode in a vertical slab gel electrophoresis apparatus (Hoefer Pharmacia Biotech Inc., CA, USA). The glass plates were treated by Repel-Silane and Binding-Silane, respectively (Dingguo, China), following the manufacturer's instructions. The system of electrophoresis was prerun in 1×TBE buffer about 30 minutes for 40°C–45°C of the gel surface temperature at 1500 v. Then the samples were separated about 4 h of migration until the bromophenol blue reached the bottom of the gel. The cDNA bands were visualized by silver staining according to Bassam et al. [23].

### 2.5. Isolation, Cloning, and Sequencing of Differential Fragments

Fragments of interest were eluted from silver-stained gels using the procedure of Frost and Guggenheim [24] with modifications. The band was eluted in 50 *μ*L of sterile double-distilled water initially at 95°C for 15 min and then hydrated overnight at 4°C. Amplifications were performed with appropriate primers with 5 *μ*L of this solution. PCR reactions were initiated at 94°C for 3 min followed by 35 cycles at 94°C for 30 s, 56°C for 30 s, 72°C for 1 min, and terminated at 72°C for 10 min. The PCR products were resolved in a 2% 1×TBE agarose gel, and each single band was isolated and eluted using the DNA Fragment Quick Purification Kit (Dingguo, China). Cloning was performed from fresh PCR products with the pGEM-T Easy vector (Promega) according to the manufacturer's instructions and using chemical transformation of one shot* E. coli* (DH5*α*) competent cells using Ampicillin as the selecting agent. After plasmid purification using a small-scale plasmid DNA purification kit (QIAGEN), the insert size was checked by PCR amplification using the corresponding selective amplification primer and the clones were sequenced by Invitrogen biotechnology service company (Shanghai, China). 

### 2.6. Real-Time PCR Analysis

RNA extraction was performed according to the above mention. Total RNA was then purified with DNase I Kit (Invitrogen, China) according to the manufacturer's instructions. First-stranded cDNAs were synthesized using PrimeScript RT Master Mix Perfect Real Time Kit (TaKaRa, China). These cDNAs were used for PCR experiments using gene-specific primers designed with DNAMAN software. Forward and reverse primers were used for producing a single amplification for the following genes: *Os04g0429600* (5′-AGGTGCCGCAGAGTTTCTAC-3′ and 5′-TACAGTGGAAGCAACCCGTTC-3′); *Os06g0124900* (5′-CCCTCTTCGTCGTCTCCAAT-3′ and 5′-TAAGCCTCTCACCTTCACGG-3′), *SRP14* (5′-CGTTTCTGAGCGAGTTGACG-3′ and 5′-GTTCTTCTTGCCATCGGTGG-3′); *Os02g0611200* (5′-TCGGCTACAGCATTGAGGAC-3′ and 5′-GGAAGAAAGGAAGCAGGACTGA-3′); *Os02g0788800* (5′-TCGGAGTTTAGAGGAGTTGCG-3′ and 5′-TAAGAGCCATCACGAGACCG-3′). qRT-PCR experiments were performed in ABI 7500 real-time PCR system and 7500 Software v 2.0.4. All PCR reactions were mixed as follows: 2 *μ*L of diluted cDNA, 10 *μ*L of 2×SYBR Premix Ex Taq II (TaKaRa, China), 0.4 *μ*L 50× ROX Reference Dye II, and 400 nmol/L of primers in a final volume of 20 *μ*L PCRs. All reactions were repeated four times. As an internal standard, a fragment of rice *actin* gene (5′-CTTCATAGGAATGGAAGCTGCGGGTA-3′ and 5′-CGACCACCTTGATCTTCATGCTGCTA-3′) was used. All the PCRs were performed under the following program: 30 s at 95°C, followed by 40 cycles of 5 s at 95°C and 34 s at 60°C in 96-well optical reaction plates (Applied Biosystems, USA).

### 2.7. High-Performance Liquid Chromatography (HPLC) Analysis of Free Polyamines

Rice leaves (1.0000 g) were weighed and crushed to homogenate using precooled marble pestle and mortar with 4 mL 5% perchloric acid. The homogenate was then suspended at 4°C for 1 h and centrifuged at 14000 g for 30 min. Supernatant was collected for the following HPLC separation and identification. All standards and polyamines extracts of spermidine, spermine, and putrescine were analyzed on an Agilent 1200 HPLC system (Agilent Corp, USA). Chromatographic separation and collection of extracts were achieved using an Eclipse Plus C18 reversed-phase column (250 mm × 4.6 mm, 5 mm; Agilent). The condition for HPLC separation was determined to be a mobile phase composed of methanol and water (75 : 25, v/v) and a flow-rate of 1 mL per min. The volume of sample injected was 10 *μ*L for the qualitative evaluation, the detection wavelength and the column temperature were set at 254 nm and 30°C, respectively. Quantification of spermidine, spermine, and putrescine in the product was determined by comparison with external standards.

### 2.8. Analysis of Sequences and Data Analysis

The sequences were analyzed for their homology against the publicly available nonredundant genes/ESTs/Transcripts in the database (http://www.ncbi.nlm.nih.gov/BLAST) using the BLASTN and BLASTX algorithms [25]. The data was analyzed for variance using the SAS/STAT statistical analysis package (version 6.12, SAS Institute, USA). Means were tested by Duncan's test at the *P*_0.05_ level.

## 3. Results 

### 3.1. Morphological Responses of Rice Seedlings to High-Temperature Treatment

**Figure 1:**
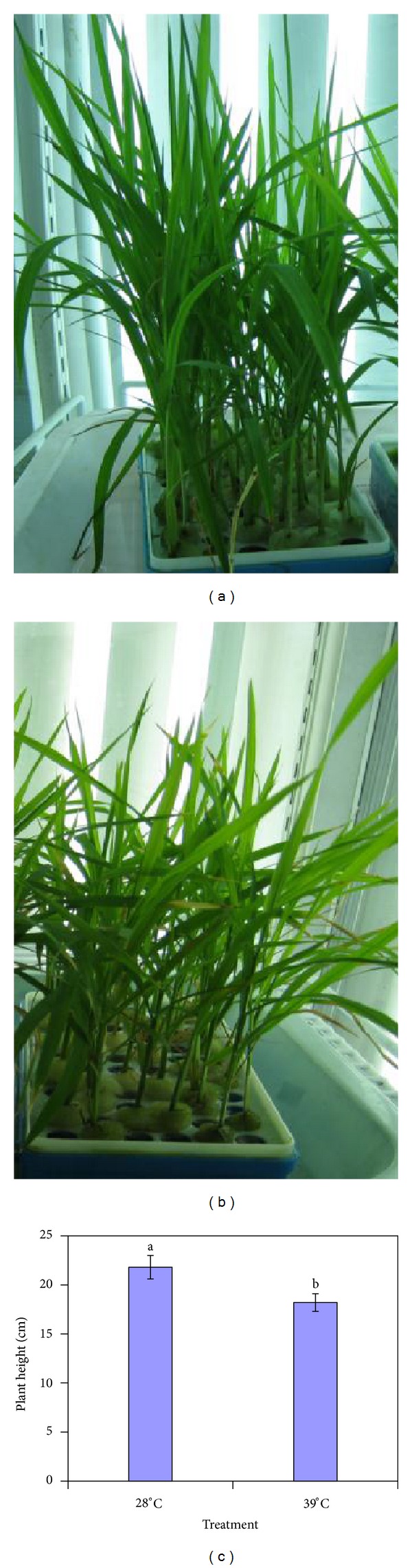
Behavior of rice seedlings (Shuanggui 1). (a) 28°C day/22°C night treatment; (b) 39°C day/30°C night treatment; (c) The comparison of plant height after 48 h at 39°C. Data from plant heights are means of three replications (50 plants per replication) ± SE. Dissimilar letters above bars differ significantly at *P* ≤ 0.05.

20-day-old seedlings were heat-stressed at 39°C day/30°C night for two days. After 48 h, the morphologies of the leaves were compared among these seedlings treated at 39°C with those treated at 28°C. As shown in Figure 1, at 28°C leaves developed normally and showed deep green (Figure 1(a)). However, conspicuous symptom has been appeared under high temperature: low-growing leaves with yellow color characters have been appeared in the heat stress treatment (Figure 1(b)). The average height of plant with 28°C treatment was 21.8 ±1.25 cm, while 39°C heat-stressed plants showed 18.2 ± 0.95 cm high, which was much lower than those of 28°C-treated rice leaves (Figure 1(c)). 

### 3.2. Identification of High-Temperature-Regulated Transcripts

In order to further understand the response of rice seedlings to heat stress, cDNA-AFLP gels of gene bands from rice leaves after treatment for 48 h at 39°C treatment were compared with those reduce extra space at 28°C treatment. Accurate gene expression profiles were determined by visual observation and analysis of band intensities, and subtle differences in transcriptional activity were revealed. After cDNA-AFLP, average 72.4 TDFs per pair of primers could be reproducibly detected mainly at 28°C while a total of average 50.5 TDFs per pair of primers changed in abundance in response to 39°C (Table 1). Therefore, on average, 61 bands (TDFs) were produced with each primer combination, which yielded more than seven thousand TDFs from seedling leaves both under control (28°C) and heat stress (39°C) treatments. A total of 145 TDFs were isolated from the silver-stained cDNA-AFLP gels based on their presence/absence (qualitative variants) or difference in the levels of expression (quantitative variants) (see Figure S1 in Supplementary Material available online at doi: http://dx.doi.org/10.1155/2013/576189). 

 Compared with the genomics profile at 28°C, average 15.9 TDFs per pair of primers were upregulated under 39°C treatment. However, compared with the genomics profile at 39°C, an average of 37.3 TDFs per pair of primers were upregulated under 28°C condition (Table 1, Figure S1), and more than three thousand differential expressing fragments were indentified. Thus, the quantity of both upregulated or downregulated cDNA fragments increased with increased temperature. This implied that the higher the temperature, the more rice life processes are affected. 

### 3.3. Changes of Rice Leaf Genomics Profile Under High-Temperature Stress

With the 64 primer combinations tested, 65 reproducible (i.e., same expression profile in 2009 and 2010 seasons) TDFs were identified, and 49 of these were cloned and sequenced. 49 differential expressed gene sequences were performed according to BLASTN (score ≥ 50) and BLASTX (E ≤ 10^−5^) analyses in NCBI and Gene Ontology databases. According to MIPS (http://mips.helmholtz-muenchen.de/proj/funcatDB/search_main_frame.html) database, differential expressed genes in leaves suffered from heat stress were classified functionally as metabolism (including carbohydrate metabolism, protein metabolism, polyamine metabolism, amino acid metabolism, ribonucleotide metabolism, and cellulose synthesis), material transport, stress response, cell cycle and fate, signal transduction, and unclear functional protein. The sequence comparison against the database revealed that most of them could be sorted into the following ten functional groups (Table 2). 

The most abundant groups were related to carbohydrate metabolism. Fourteen TDFs response to the heat stress were identified as related to carbohydrate metabolism. Among them, four TDFs (H51-4, H53-1, -2, and H57-3, Figure S1 and Table 2) corresponding to photosynthesis II-related protein, NADH dehydrogenase subunit 2, chloroplast ATP synthase a chain precursor, and chloroplast ATP synthase a chain precursor, respectively, were upregulated by heat stress treatment (Figure S1 and Table 2), while two TDFs (N21-1, N58-3), one of them related to photorespiration proteins (NAD-dependent epimerase/dehydratase family protein) and the other corresponding to phospho-2-dehydro-3-deoxyheptonate aldolase 1, were downregulated (Table 2). Two TDFs (H1-5, H51-5) corresponding to ubiquitin family protein and 60S ribosomal protein, which were sorted into protein metabolism group, were also upregulated by the heat stress (Table 2). One identified TDF (H57-5) corresponding to S-adenosylmethionine decarboxylase, which has been classified into polyamine metabolism, was also affected by heat stress treatment. To further validate the function of S-adenosylmethionine decarboxylase in rice seedling leaves, we used HPLC to investigate the effect of heat stress on the concentration of spermidine, spermine, and putrescine in rice seedling leaves. As shown in Figure 3, spermidine, spermine, and putrescine contents were increased dramatically at 39°C compared with those at 28°C. In addition, one TDF (H51-1) corresponding to glutamate decarboxylase, which could be sorted into amino acid metabolism, has also shown increasing trend under heat stress treatment. Two upregulated heat stress-induced differential fragments (H3-2-8, H46-3), which are corresponding to CTP synthase and cellulose synthesis family protein, were classified into ribonucleotide metabolism and cellulose metabolism, respectively. There are five heat-stressed induced genes identified and classified into material transport group: 2 (H51-3, H59-4) of 3 corresponding to phophate translocator and amino acid/polyamine transporter II were upregulated at 39°C (Table 2). Other two TDFs (H53-8, N60-4) corresponding to thioredoxin h isoform 1 and heat shock protein (HSP) DnaJ, respectively, could be sorted into stress response group. One upregulated heat stress-induced differential fragment (H4-1) and the other downregulated fragment (N6-1), which are corresponding to S-phase-specific ribosomal protein and putative senescence-associated protein, were classified into cell cycle and fate group (Table 2). Other gene fragments regulated with 39°C treatment included sixteen gene bands corresponding to hypothetical proteins (H3-2-1, H51-2, N60-5, etc.), whose functions were unknown or unclear (Table 2 and Figure S1).

### 3.4. Differential Response of Gene Fragments to Heat Stress

**Figure 2:**
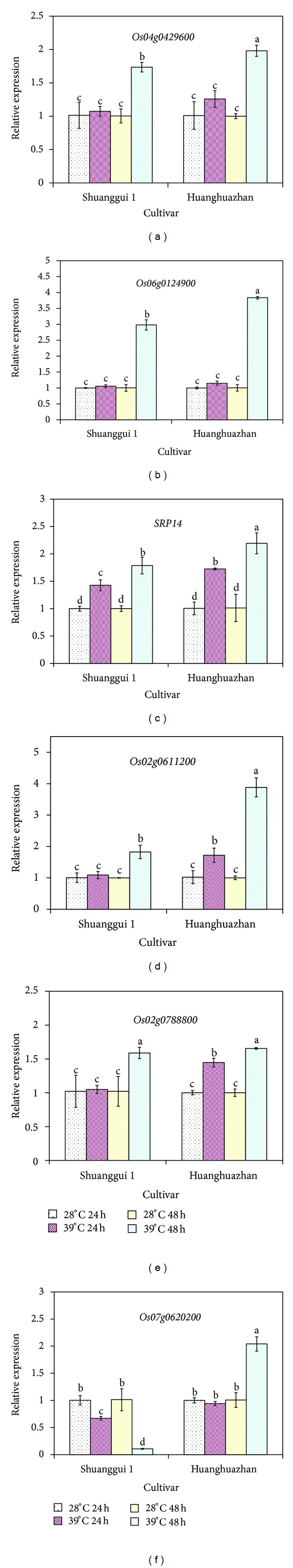
Relative expression of *Os04g0429600*, *Os06g0124900*, *SRP14*, *Os02g0611200*, *Os02g0788800*, and *Os07g0620200* compared to that of *actin* detected by real-time quantitative RT-PCR in plants at 28/22°C or plants at 39/30°C for 24 hours and 48 hours in heat-sensitive Shuanggui 1 and heat-tolerant Huanghuazhan. Values indicate relative expression levels against those of the same genes at 28/22°C in three biological replications from cDNA prepared from leaves. *Os04g0429600*, cellulose synthase-like protein H1; *Os06g0124900*, hypothetical protein; *SRP14*, signal recognition particle subunit 14; *Os02g0611200*, S-adenosylmethionine decarboxylase proenzyme (AdoMetDC) (SamDC); *Os02g0788800*, amino acid/polyamine transporter II; *Os07g0620200*, heat shock protein DnaJ, N-terminal domain containing protein.

To validate the results of cDNA-AFLP experiment and quantitatively assess the relative abundance of the transcripts in rice seedling leaves under the heat stress, five upregulated TDFs (H46-3,* Os04g0429600*; H51-2,* Os06g0124900*; H51-6, *SRP14*;H57-5,* Os02g0611200*, and H59-4,* Os02g0788800*) and one downregulated TDF (N60-4,* Os07g0620200*) were selected for RNA expression analysis. Initially, a semiquantitative RT-PCR was performed to analyze the changes in transcripts of all six TDFs in response to the heat stress (data not shown). Obtained RT-PCR results were substantiated with quantitative real-time PCR (qRT-PCR) (Figure 2). Rice *actin* gene was selected as internal control for normalization.

The transcript of all six genes fragments (*Os04g0429600*,* Os06g0124900*, *SRP14*,* Os02g0611200*, *Os02g0788800*, and *Os07g0620200*) responded to heat stress (Figure 2). The most pronounced effect was observed in the case of *Os06g0124900* gene which increased more than twice after 48 h at 39°C treatment both in heat-sensitive “Shuanggui 1” and heat-tolerant “Huanghuazhan” and more increasing trend could be observed in heat-tolerant cultivar, though there was no obvious change after 24 h with 28°C treatment. Fast transcript accumulation was observed in case of *SRP14* and *Os07g0620200* with heat stress. *SRP14 *showed significant increase in transcript accumulation only after 24 h at 39°C compared with that with same time at 28°C and relative expression of this gene presented more increasing trend in heat-tolerant cultivar compared to that in heat-sensitive cultivar. Fast downregulated trend has been observed in *Os07g0620200* gene, which has also happened only after 24 h heat stress treatment in heat-sensitive cultivar and considerable decreasing presented has been shown after 48 h under heat stress condition. However, fast effect of heat stress did not take place in heat-tolerant cultivar with no considerable variation after 24 h treatment both at 28°C or 39°C while obvious transcript increase could be observed after 48 h of heat stress treatment. The transcripts of *Os04g0429600*, *Os02g0611200*, and *Os02g0788800* also showed up-regulation with no more than twofold due to heat stress with no significant change after 24 h and gradual increase after 48 h. In general, five TDFs (*Os04g0429600*,* Os06g0124900*, *SRP14*,* Os02g0611200*, and *Os02g0788800*) expressions were increased under heat stress with one TDF (*Os07g0620200*) shown downregulated under the same condition. 

## 4. Discussion

Previous genomic and proteomic studies on rice responses to heat stress have provided some valuable results. Genes [26] and proteins [27] of rice grain under heat stress conditions have been identified. Changes of proteomic profiles in rice seedling leaves at high temperature [4] have also been reported. In this study, responses of rice seedlings to heat stress environment (39°C day/30°C night) were investigated. Among TDFs, which were responsive to 39°C, 49 of them have been identified. The corresponding proteins or relative nucleotides of these identified TDFs were related to energy (photosynthesis and photorespiration) transportation, transcription, or translation, and other biological functions, respectively. Furthermore, unlike previous transcriptomic [26] and proteomic [4, 9, 27] studies, in our study 49 TDFs were identified as high-temperature-responsive genes. The identified genes provided valuable information by which the tolerance mechanisms of rice exposed to heat stress can be unveiled in detail.

### 4.1. Effect of Heat Stress on the Biological Process of the Rice Seedling

In this study, there are fourteen differential expression fragments, which were classified into carbohydrate metabolism group. The upregulated TDF (H51-4, Table 2) in response to heat stress is the chloroplast gene (*psbH*), which encodes a 9-10 kDa thylakoid membrane protein (PSII-H). PSII protein H is associated with photosysthem II and is subject to light-dependent phosphorylation at a threonine residue located on the stromal side of the membrane [28]. Thus, the up-regulation of *psbH* gene could protect the photosynthetic machinery in the high-temperature-stressed rice seedlings. Other two differential expression genes (N21-1, H53-1, Table 2), which are involved in carbohydrate metabolism, are also induced by the heat stress. TDF (N21-1, Table 2) corresponding to NAD dependent epimerase/dehydratase family protein is downregulated under 39°C treatment; TDF (H53-1, Table 2) corresponding to NADH dehydrogenase subunit 2 is upregulated by the stress treatment. NAD-dependent epimerase/dehydratase family protein takes part in the carbohydrate metabolic biological process, which includes the formation of carbohydrate derivatives by the addition of a carbohydrate residue to another molecule. Hence, downregulated TDF (N21-1, Table 2) suggested that heat stress may inhibit the carbohydrate derivatives formation in the seedling stage of seedling leaves. NADH dehydrogenase, which is responsible for the oxidative phosphorylation [29], is so-called entry-enzyme of the mitochondrial electron transport chain. The up-regulation of NADH dehydrogenase indicated that protective mechanism involved in electron transportation process may be stimulated in the heat-stressed rice seedling. In spite of down-regulation of NAD-dependent epimerase involved in carbohydrate derivatives formation, the more significant electronic transportation process regulated by NADH dehydrogenase showed upregulated trend indicating the protective capability of rice seedling confronted with heat stress. 

In cellulose metabolism category, TDF (H46-3, Table 2 and Figure S1), which encodes cellulose synthase family protein, was also upregulated by the heat stress stimulation. The transcript expression of this enzyme in cDNA-AFLP was also correlated with the results in real-time RT-PCR (Figure 2). Earlier work concluded that cellulose synthase is the key enzyme involved in cellulose synthesis in cell wall [30]. The up-regulation of cellulose synthase during the heat stress indicated that high-temperature may improve the formation of cell wall and, hence, assumed the protective function against the damage generated by the temperature for the rice seedling. 

Besides the most abundant groups mentioned above, there were still other TDFs, which were identified as functions involved in material transportation, cell structure and cycle, polyamine metabolism, and signal transduction, and so forth. In spite of fewer TDFs found in these groups, the accordance of the semiquantitative RT-PCR results (data not shown) and the real-time PCR results (Figure 2) with the cDNA-AFLP analysis (Figure S1) indicated that these fragments with corresponding functional groups were also affected severely by the increase of the temperature. Therefore, the identification of the TDFs will be a solid foundation for the future researches. 

 Five genes, were which, involved in material transportation were increasely expressed under heat stress, which implied a temperature-related activation of this process. In this study, TDF (H59-4, Table 2), identified as amino acid permease, which could import serine and generate sphingoid bases during heat stress [31], was significantly upregulated at 39°C treatment. The activity change of TDF (H59-4), agreeing well with the change of mRNA expression of amino acid permease (Figure 2), confirmed that permease-regulated amino acid uptake could be increased by the stimulation of the heat stress. In addition, another TDF (H51-3, Table 2 and Figure S1) corresponding to triose phosphate translocator protein could also be included into material transporters group. Triose phosphate translocator protein is a membrane protein responsible for exchanging all carbohydrate products produced in photosynthesis in plants and, therefore, could mediate the export of fixed carbon in the form of triose phosphates and 3-phosphoglycerate from the chloroplasts into the cytosol [32]. So the up-regulation of TDFs (H51-3) indicated that the increase of the temperature could promote the carbohydrate products transportation produced in photosynthesis and, hence, provide the protective mechanism for the rice seedling leaves during the heat stress.

**Figure 3:**
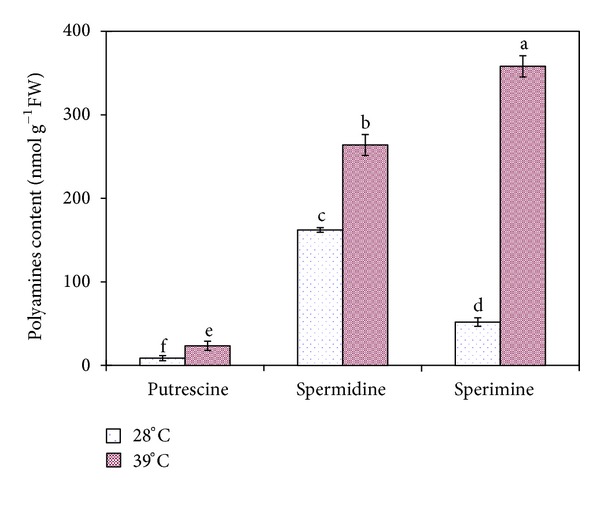
Variation of levels of free spermidine, spermine, and putrescine in rice seedlings under 39°C and 28°C treatments with HPLC analysis. Data from levels of free spermidine, spermine, and putrescine in rice seedlings are means of three replications ± SE. Dissimilar letters above bars differ significantly at *P* ≤ 0.05.

There are other two upregulated TDFs (H51-6, Figure S1 and Table 2; H57-5, Figure S1 and Table 2), which encode signal recognition particle subunit and S-adenosylmethionine decarboxylase, classified into two newly functional groups: signal transduction and polyamine metabolism. The up-regulation of TDF (H51-6, Figure S1 and Table 2), which encodes signal recognition particle subunit, suggested that high temperature could also improve the signal transduction process during the heat stress. S-Adenosylmethionine decarboxylase is an enzyme whose capability is to catalyze the conversion of S-adenosyl methionine to S-adenosylmethioninamine. S-adenosylmethionine decarboxylase plays an essential regulatory role in the polyamine biosynthetic pathway by producing the n-propylamine residue required for the synthesis of spermidine and spermine from putrescine [33, 34]. Spermidine and spermine synthase (SPDS; EC 2.5.1.16 and SPMS; EC 2.5.1.22) could synthesize higher spermidine and spermine by the successive addition of aminopropyl groups to putrescine [35]. Our result has also proved that the upregulated expression of S-adenosylmethionine decarboxylase gene agreed well with the contents of spermidine and spermine (Figures 2 and 3). In addition, one TDF (N60-4, Table 2, Figure S1) corresponding to heat shock protein (hsp) has been classified into stress response group. Its down-regulation function, which was agreeing well with the real-time RT-PCR result (Figure 2), indicated that the gene was affected obviously under heat stress condition. 

In conclusion, this study reported 49 identified differential expression fragments in rice seedlings in respons to the heat stress. Meanwhile, ten functional groups classification containing 49 TDFs indicating different strategies were employed by rice seedlings exposed to different temperatures: the higher the temperature, the more effects could be observed. Our future research could be focused to reveal functions of the genes indentified under heat stress.

## Supplementary Material

A representative photograph of a silver-stained cDNA-AFLP gel.

## Figures and Tables

**Table 1:** Summary of PAGE showing bands after 64 pairs of primers amplification (per pair of primes).

Treatment	Average number of amplified gene bands	Average number of upregulated and nascent fragments
28°C	72.4	37.3
39°C	50.5	15.9

28°C : 28°C (day)/22°C (night); 39°C : 39°C (day)/30°C (night).

**Table 2:** The nucleotide-homology of the transcript-derived fragments (TDFs) with known gene sequences in the database using the BLASTN and BLASTX algorithms along with their expression patterns.

TDF no.	TDF size (bp)	GenBank/gene	Corresponding or related protein	Score (bits)	Identities	*E* value
Carbohydrate metabolism						

N21-1	130	*ABF96013.1 *	NAD-dependent epimerase/dehydratase family protein	73.2 (178)	32/33 (97%)	2*e* − 14*
H50-1	195	*PsaB *	PSI P700 apoprotein A2	200 (108)	108/108 (100%)	3*e* − 48
H51-4	198	*YP_654233 *	PS II protein H	57.8 (138)	26/27 (96%)	2*e* − 09*
H51-8	138	*ABI34757.1 *	Ribulose-1,5-bisphosphate carboxylase/oxygenase large subunit (*Pentameris aspera*)	80.5 (197)	34/35 (97%)	3*e* − 17*
H53-1	495	*NP_039432.1 *	NADH dehydrogenase subunit 2	205 (522)	139/171 (81%)	1*e* − 60*
H53-2	389	*Os10g0527100 *	Chloroplast ATP synthase a chain precursor	217 (117)	119/120 (99%)	6*e* − 53
H53-3	299	*Os08g0472600 *	Alpha-1, 3-fucosyltransferase	152 (82)	87/89 (98%)	1*e* − 33
H53-6	261	*Os10g0527100 *	Chloroplast ATP synthase a chain precursor	433 (234)	236/237 (99%)	4*e* − 118
H53-7	220	*Os07g0662900 *	4-Alpha-glucanotransferase	76.8 (41)	46/48 (96%)	6*e* − 11
H56-3	229	*AAA84588.1 *	atpB gene product	145 (366)	71/76 (93%)	1*e* − 39*
H57-3	294	*Os10g0527100 *	Chloroplast ATP synthase a chain precursor	224 (121)	123/124 (99%)	3*e* − 55
H57-4	290	*Os10g0527100 *	Chloroplast ATP synthase a chain precursor	224 (121)	123/124 (99%)	3*e* − 55
H57-6	195	*Os01g0881600 *	Photosystem II reaction center J protein	110 (59)	61/62 (98%)	5*e* − 21
N58-3	270	*Os08g0484500 *	Phospho-2-dehydro-3-deoxyheptonate aldolase 1, chloroplast precursor	207 (112)	116/118 (98%)	3*e* − 50

Protein metabolism						

H1-5	227	*Os03g0131300 *	Ubiquitin domain containing protein	283 (153)	159/162 (98%)	3*e* − 73
H51-5	185	*AF093630 *	60S ribosomal protein L21 (RPL21)	298 (161)	168/171 (98%)	1*e* − 77
N53-6	294	*Os07g0555200 *	Eukaryotic translation initiation factor 4G	147 (79)	82/83 (99%)	6*e* − 32

Polyamine metabolism						

H57-5	260	*Os02g0611200 *	S-adenosylmethionine decarboxylase proenzyme (AdoMetDC) (SamDC)	169 (91)	96/98 (98%)	1*e* − 38

Amino acid metabolism						

H51-10	118	*GAD *	Glutamate decarboxylase	172 (93)	103/107 (96%)	3*e* − 40

Ribonucleotide metabolism						

H3-2-8	102	*Os12g0556600*	CTP synthase family protein	143 (77)	79/80 (99%)	2*e* − 31

Cellulose synthesis						

H46-3	193	*Os04g0429600*	Cellulose synthase-like protein H1	124 (67)	100/115 (87%)	2*e* − 25

Material transport						

N51-17	181	*Os12g0166000 *	Peptidase S59, nucleoporin family protein	189 (102)	111/115 (97%)	6*e* − 45
H51-3	238	*Os01g0239200 *	Phophate translocator	399 (216)	218/219 (99%)	3*e* − 108
N53-1	372	*Os08g0517200 *	Ca^2+^-ATPase isoform 9	580 (314)	316/317 (99%)	2*e* − 162
H57-2	304	*Os02g0176700 *	Potential calcium-transporting ATPase 9, plasma membrane type	505 (273)	282/286 (99%)	9*e* − 140
H59-4	276	*Os02g0788800 *	Amino acid/polyamine transporter II family protein	466 (252),	252/252 (100%)	4*e* − 128

Stress response						

H53-8	191	*Os07g0186000 *	Thioredoxin h isoform 1	189 (102)	104/105 (99%)	6*e* − 45
H53-9	186	*Os07g0186000 *	Thioredoxin h isoform 1	187 (101)	104/105 (99%)	2*e* − 44
N60-4	307	*Os07g0620200 *	Heat shock protein DnaJ, N-terminal domain containing protein	403 (218)	218/218 (100%)	3*e* − 109

Signal transduction						

H51-6	175	*SRP14 *	Signal recognition particle Subunit 14	279 (151)	156/158 (99%)	3*e* − 72
H51-7	144	*SRP14 *	Signal recognition particle Subunit 14	209 (113)	122/126 (97%)	3*e* − 51

Cell cycle and fate						

H4-1	357	*RSPSP94 *	S-phase-specific ribosomal protein	604 (327)	329/330 (99%)	1*e* − 169
N6-1	296	*AY436773.1 *	Putative senescence- associated protein (*Pyrus communis*)	503 (272)	277/279 (99%)	3*e* − 139

Unclear functional proteins						

H3-2-1	200	*Os05g0156800 *	Hypothetical protein	263 (142)	144/145 (99%)	4*e* − 67
N19-2	369	*BAH80065.1 *	Putative retrotransposon protein	173 (438)	97/100 (97%)	4*e* − 48*
N51-2	436	*Os12g0597400 *	Hypothetical protein	758 (410)	414/416 (99%)	0.0
N51-3	411	*Os12g0444500 *	Hypothetical protein	479 (259)	270/275 (98%)	8*e* − 132
N51-4	399	*BAD68598.1 *	Hypothetical protein	46.6 (109)	29/59 (49%)	5*e* − 04*
H51-2	250	*Os06g0124900 *	Hypothetical protein	265 (143)	179/194 (92%)	1*e* − 67
H51-9	128	*Os01g0750800 *	Hypothetical protein	174 (94)	102/106 (96%)	1*e* − 40
N53-5	298	*AAQ56570.1 *	Polyprotein	145 (367)	79/125 (63%)	1*e* − 41*
H53-11	143	*Os04g0599650 *	Tetratricopeptide-like helical domain containing protein	230 (124)	139/145 (96%)	3*e* − 57
H53-12	140	*Os04g0599650 *	Tetratricopeptide-like helical domain containing protein	228 (123)	133/137 (97%)	9*e* − 57
H55-2	169	*Os01g0795000 *	Hypothetical protein	274 (148)	148/148 (100%)	1*e* − 70
H56-4	212	*Os03g0685500 *	Hypothetical protein	172 (93)	93/93 (100%)	7*e* − 40
H56-5	151	*Os12g0590400 *	Hypothetical protein	147 (79)	91/96 (95%)	3*e* − 32
H56-8	173	*Os03g0395600 *	Hypothetical protein	204 (110)	110/110 (100%)	2*e* − 49
N60-5	303	*EEC84589.1 *	Hypothetical protein OsI_31400	90.9 (224)	45/48 (94%)	5*e* − 19*
N62-8	305	*AAT44171.1 *	Hypothetical protein	73.2 (178)	35/36 (97%)	1*e* − 15*

The “H” before the numbers of the TDFs represents upregulated expression in high-temperature treated plants, while “N” represents upregulated expression in normal temperature treated plants (downregulated gene under high temperature). All are BLASTN scores except for those marked with “*”, which are BLASTX scores.
